# Reliability of Three Disability Scales for Detection of Independence Loss in Parkinson's Disease

**DOI:** 10.1155/2016/1941034

**Published:** 2016-04-24

**Authors:** Anders Bjornestad, Ole-Bjorn Tysnes, Jan Petter Larsen, Guido Alves

**Affiliations:** ^1^The Norwegian Centre for Movement Disorders, Stavanger University Hospital, 4068 Stavanger, Norway; ^2^Institute of Clinical Medicine, University of Bergen, 5021 Bergen, Norway; ^3^Department of Neurology, Stavanger University Hospital, 4068 Stavanger, Norway; ^4^Department of Neurology, Haukeland University Hospital, 5021 Bergen, Norway

## Abstract

*Background.* Loss of independence is considered an important outcome measure in Parkinson's disease (PD), but tools to assess dependency have not been tested in PD.* Methods.* In this study of 158 PD patients, we examined the two most widely used scales and cut-offs for dependency evaluation in PD, the Hoehn and Yahr (HY) stage > 3 and the Schwab and England (SE) scale score < 80%, against a standardized clinical interview assessing dependency in activities of daily living (ADL). We also examined the performance of the generic Barthel ADL index. In addition, we determined whether alternative cut-offs improved the utility of these tools.* Results.* Compared to clinical interview as gold standard, HY stage > 3 had 21% sensitivity and 98% specificity in detecting dependency in ADL. Corresponding figures for SE score < 80% were 55% and 92%, respectively. Using alternative cut-off values improved the overall diagnostic accuracy only slightly. Barthel ADL index had 67% sensitivity and 78% specificity in detecting dependency at its optimal cut-off value.* Conclusion.* Both the disease-specific HY staging and SE scale and the generic Barthel ADL index are suboptimal tools for assessing independence loss in PD. Clinical interview should be the assessment of choice in studies of dependency.

## 1. Introduction

The ability to live independently is an important determinant of quality of life [1]. Loss of independence in basic activities of daily living (ADL) such as administration of medication, dressing, personal hygiene, eating, and house chores or needing admission to a care facility might be the first sign of increasing disability and declining functional status and is therefore considered a crucial event in the progression of Parkinson's disease (PD). However, frequency estimates of independence loss vary substantially in PD, and little is known about associated risk factors [2]. In order to conduct informative studies on this subject, sensitive and specific assessment tools to detect loss of independence are necessary. Furthermore, reliable generic instruments are needed to allow estimation of the risk of losing independence in PD relative to nonaffected elderly people.

A recent systematic review [2] reported that the tool most frequently used to assess loss of independence in PD is Hoehn and Yahr staging [3], with a score exceeding 3 defining dependency in most studies. The Schwab and England ADL scale [4], with scores below 80% defining dependency, has also been used. However, these scales were designed to assess disability, have not been tested as tools to detect dependency [5], and are both PD-specific. The generic 10-item Barthel ADL index [6] might be a better measurement of dependency than the other two scales [2]. However, it is less used and has not been fully validated in PD [7].

As there is uncertainty regarding the optimal assessment tools for independence loss in PD, we performed a clinimetric study in a large PD cohort to test the reliability of these instruments in detecting dependency compared to a standardized clinical interview as the gold standard.

## 2. Methods

### 2.1. Subjects

All subjects participate in the Norwegian ParkWest study, a prospective, community-based, longitudinal study of patients with incident PD designed to investigate the incidence, neurobiology, and prognosis of the disease [8]. For this clinimetric study, we included all 158 patients who attended the 5-year follow-up visit, as examinations at this point included both clinical interview and the different scales described below. All subjects met widely acknowledged diagnostic research criteria of PD [9, 10]. The study was approved by the Regional Committee for Medical and Health Research Ethics, Western Norway. Signed written consent was obtained from all participants.

### 2.2. Assessments

All examinations were performed by neurologists experienced in movement disorders. A wide array of clinical and demographic variables were assessed, including age, gender, disease duration, motor severity using the Unified PD Rating Scale [11], depressive symptoms as assessed by the Montgomery-Åsberg Depression Rating Scale [12], global cognition as measured by the Mini-Mental State Examination [13], and dementia status according to Movement Disorders Society criteria [14], as described previously [15].

Disease stage was evaluated according to the modified Hoehn and Yahr (HY) staging [16], a measure of both impairment and disability that ranges from 0 (no visible symptoms of PD) to 5 (wheelchair bound or bedridden unless aided). Disability was assessed according to the Schwab and England (SE) ADL scale [4], ranging from 100% (completely independent, essentially normal) to 0% (bedridden, vegetative function, completely invalid). Furthermore, the 10-item Barthel ADL index (BI) [17] was employed as an auxiliary test of dependency. The BI sum score ranges from 0 to 20, higher scores indicating more independent functioning. In the same session, a standardized clinical interview with patients and their caregivers was performed to assess dependency status in basic ADL. The interview addressed living situation (at home or in intermittent or continuous facility care) and, if living at home, the source (e.g., friends, relatives, cleaning or food delivery personnel, and community nurses) and type (e.g., administration of medication, dressing, personal hygiene, eating, house chores, and general supervision) of help received. Loss of independence was defined as receiving regular help with basic ADL, regardless of location, source, and type.

### 2.3. Statistical Analysis

IBM SPSS version 22.0 was used for statistical analyses. We first determined the diagnostic performance of the most widely used cut-offs of the HY staging (stage > 3) and SE scale (score < 80%) in detecting dependency in basic ADL. We subsequently generated receiver operating characteristic (ROC) curves to calculate the area under the ROC curve (AUC) for these scales and cut-offs and used Youden's *J*-statistic to evaluate whether more appropriate cut-off levels existed. We also explored the reliability of the BI using its optimal sum score cut-off, as determined by ROC analysis and Youden's *J*-statistic. Cohen's unweighted kappa values interpreted according to Landis and Koch [18] were used to evaluate the reliability and concordance of the tools. Analyses were run in the overall sample (*n* = 158) and separately in nondemented (*n* = 132) patients. Excluding demented patients did not improve analytic performance of the disability scales; therefore, results from the overall sample are presented.

## 3. Results

The demographic and clinical characteristics of the 158 patients included in this clinimetric study are provided in Table 1. Of the 158 patients, 58 (36.7%) reported dependency in basic ADL during the clinical interview. Among dependent patients, 28 received help from non-healthcare professionals (e.g., families, friends, and cleaning or food delivery services), 16 had community nursing, 5 were in intermittent facility care, and 9 were in long-term facility care.

### 3.1. Hoehn and Yahr Staging

#### 3.1.1. Cut-Off > 3.0

**Figure 1:**
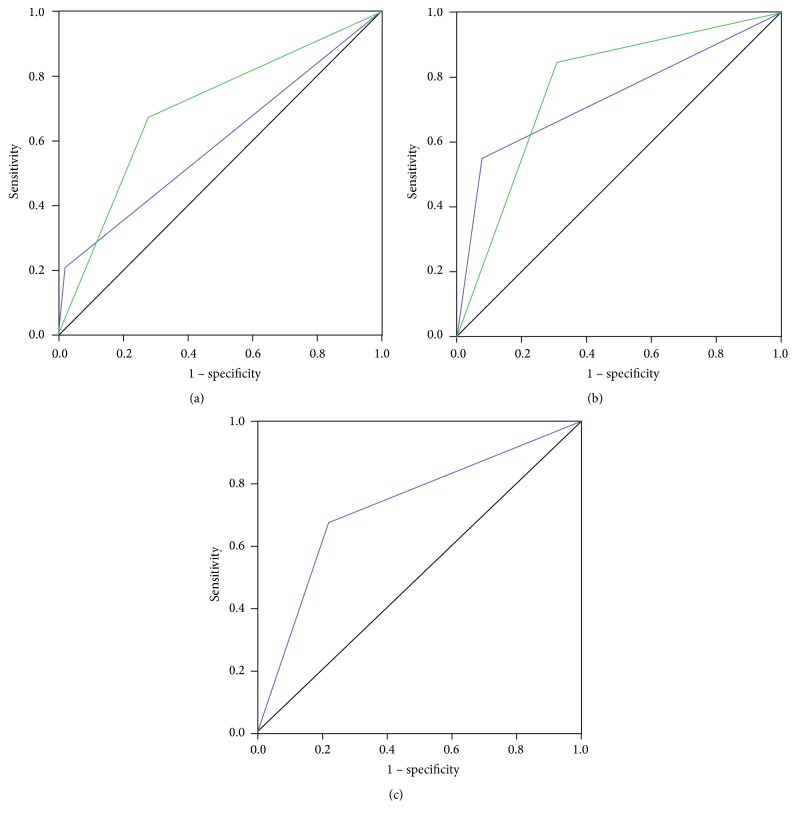
Receiver operating characteristic curves of (a) Hoehn and Yahr at cut-offs of >3.0 (blue) and >2.0 (green). (b) Schwab and England ADL scale at cut-offs of <80% (blue) and <90% (green). (c) Barthel index score at a cut-off of <20.

Of the 58 patients reporting dependency in basic ADL during the interview, 12 were in HY stage > 3. Thus, sensitivity of this cut-off to detect dependency was 20.7%. Of the 46 dependent patients missed by this cut-off, 28 received help from non-healthcare professionals, 13 had community nursing, and 2 were in intermittent and 3 in long-term facility care. Two of the 100 patients who were independent had HY > 3, yielding a specificity of 98.0%. Positive predictive value (PPV) was 85.7% and negative predictive value (NPV) was 68.1%. ROC curve analysis (Figure 1(a)) showed an AUC of 0.59 (95% CI (0.50–0.69), *p* = 0.05). Reliability was only fair compared to clinical interview (Table 2). Concordance with other scales was moderate at best (Table 3).

#### 3.1.2. Optimal Cut-Off

According to Youden's *J*-statistic, the optimal cut-off to detect dependency was HY stage > 2.0, increasing the AUC (Figure 1(a)) to 0.70 (0.61–0.78, *p* < 0.001). However, while sensitivity (67%) and NPV (79%) increased, specificity (67%) and PPV (58%) decreased. Reliability was still only fair compared to clinical interview (Table 2). Concordance with other scales was moderate (Table 3).

### 3.2. Schwab and England Scale

#### 3.2.1. Cut-Off < 80%

Thirty-two of the 58 patients reporting dependency in basic ADL had SE score < 80%, yielding a sensitivity of 55.2% (Table 2). Of the 26 dependent patients not detected, 19 received help from non-healthcare professionals, 5 had community nursing, and 1 was in intermittent and 1 was in long-term facility care. Eight of the 100 independent patients had SE score < 80%, resulting in 92.0% specificity. PPV was 80.0% and NPV was 78.0%. The AUC (Figure 1(b)) was 0.74 (0.64–0.82, *p* < 0.001). Reliability compared to clinical interview (Table 2) and concordance with other scales (Table 3) was moderate.

#### 3.2.2. Optimal Cut-Off

Youden's *J*-statistic suggested a SE score < 90% as the optimal cut-off to detect dependency, increasing sensitivity (85%) and NPV (88%), while specificity (69%) and PPV (61%) decreased (Table 2). The AUC (Figure 1(b)) at this cut-off was 0.77 (0.69–0.84, *p* < 0.001). Reliability compared to clinical interview remained moderate (Table 2). Concordance with the other scales was slight to moderate (Table 3).

### 3.3. Barthel ADL Index

To our knowledge, there is no established BI cut-off to detect dependency in PD. We found a BI sum score cut-off < 20 to be optimal, providing a sensitivity of 67%, specificity of 78%, PPV of 64%, and NPV of 80%. The AUC (Figure 1(c)) was 0.74 (0.65–0.83, *p* < 0.001), and the reliability was moderate compared to the clinical interview (Table 2). Concordance with other scales was fair to moderate (Table 3).

## 4. Discussion

In this clinimetric study, we investigated the reliability of and concordance between three disability scales in detecting loss of independence in basic ADL among patients with PD. Compared to clinical interview as the gold standard, we found that the disease-specific HY stage and SE scale were highly specific but not sufficiently sensitive when using the most widely applied cut-offs to define dependency. We also explored the reliability of the generic BI for which no cut-off for defining dependency has been established previously. However, even with a statistically optimal cut-off, the sensitivity of the BI to detect dependency in basic ADL proved suboptimal. Our data raise concerns about the reliability of these three measures as tools to assess loss of independence in PD.

Among the three scales tested in this study, the HY staging had the lowest overall accuracy in detecting dependency in basic ADL. This is striking given that this scale has been the most frequently used tool to assess loss of independence in PD so far, with a cut-off of >3 defining dependency in most studies [2]. While specificity was very high at this cut-off, sensitivity was poor, only 21%. The use of HY staging with this mainly arbitrary cut-off in research on ADL dependency in PD thus seems inappropriate, and the lack of focus on ADL in this staging system emphasizes this. Although the SE scale showed higher reliability than the HY staging, sensitivity to detect dependency at the most commonly used cut-off (score < 80%) was only 55%. This suggests that previous studies using these cut-offs most likely have provided substantial underestimates of the true frequency of independence loss in PD. In addition, we observed at best moderate concordance between these scales, which probably explains the large variability in frequency estimates of independence loss in PD [2]. These factors may also have biased the research on risk factors for dependency associated with PD.

Our analyses revealed alternative cut-offs that improved the overall accuracy of the HY staging and SE scale in discriminating between dependency and independence in basic ADL, though at the cost of reduced specificity. Still, for the SE scale, we observed both high sensitivity (85%) and NPV (88%) using the “optimal” cut-off (score < 90%), which could therefore be considered a potential screening instrument for dependency in PD, preferably followed by a clinical interview to reach maximum specificity.

Disease-specific scales such as the HY staging and the SE scale do not allow comparisons with the general population. This would, however, be important given that loss of independence in basic ADL is common in elderly people and may vary between populations due to social and cultural differences. Therefore, we also explored the reliability of the generic BI in detecting loss of independence in PD. As the disease-specific measures, the BI showed rather high specificity (78%) but suboptimal sensitivity (67%) in detecting dependency in basic ADL in our cohort.

## 5. Conclusion

Our study does not support the use of Hoehn and Yahr staging, Schwab and England scoring, or Barthel index scoring as instruments to assess dependency in basic ADL in patients with PD. Further research on this subject is needed, given that loss of independence is considered a crucial life event and therefore is an important outcome measure in assessing disease progression in PD. Until better tools are developed, our results suggest that loss of independence should be assessed by a clinical interview.

## Figures and Tables

**Table 1:** Characteristics of the 158 Parkinson's disease patients at the 5-year visit.

Characteristics	Overall	Independent	Dependent
Patients, *n*	158	100	58
Male, *n* (%)	96 (60.8)	59 (59.0)	37 (63.8)
Age, years	71.4 (9.1)	69.7 (9.5)	74.3 (7.4)
UPDRS motor score	25.2 (12.9)	20.6 (9.7)	33.2 (13.9)
Hoehn and Yahr stage	2.3 (0.8)	2.1 (0.6)	2.8 (0.9)
Stage 1.0/1.5, *n* (%)	21 (13.2)	20 (20.0)	1 (1.7)
Stage 2.0, *n* (%)	69 (43.7)	51 (51.0)	18 (31.0)
Stage 2.5, *n* (%)	32 (20.3)	19 (19.0)	13 (22.4)
Stage 3.0, *n* (%)	21 (13.2)	7 (7.0)	14 (24.1)
Stage 4.0/5.0, *n* (%)	14 (8.9)	2 (2.0)	12 (20.7)
Schwab and England score	79.1 (16.4)	85.9 (9.3)	67.2 (19.1)
Score ≥ 90%, *n* (%)	78 (49.4)	69 (69.0)	9 (15.5)
Score 80%, *n* (%)	40 (25.3)	23 (23.0)	17 (29.3)
Score < 80%, *n* (%)	40 (25.3)	8 (8.0)	32 (55.2)
Barthel ADL index	18.5 (3.4)	19.6 (1.0)	16.5 (4.8)
MADRS score	4.4 (5.0)	3.5 (4.3)	6.0 (5.7)
MMSE score	26.7 (3.9)	27.9 (2.9)	24.7 (4.6)

Values are mean (SD) if not otherwise indicated.

All differences between independent and dependent patients were significant, except for gender.

UPDRS: Unified Parkinson's Disease Rating Scale; MMSE: Mini-Mental State Examination; MADRS: Montgomery-Åsberg Depression Rating Scale.

**Table 2:** Reliability of the Hoehn and Yahr stage, Schwab and England scale, and Barthel ADL index in the detection of loss of independence in PD compared to clinical interview.

	Sensitivity	Specificity	PPV	NPV	AUC	Kappa
Hoehn and Yahr						
Stage > 3.0	21%	98%	86%	68%	0.59	0.22
Stage > 2.0^a^	67%	72%	58%	79%	0.70	0.38
Schwab and England						
Score < 80%	55%	92%	80%	78%	0.74	0.50
Score < 90%^a^	85%	69%	61%	88%	0.77	0.50
Barthel ADL index						
Score < 20^a^	67%	78%	64%	80%	0.73	0.45

^a^Optimal cut-off according to Youden's *J*-statistic and ROC curve analysis.

Kappa values exceeding 0.20 represent fair, 0.40 moderate, and 0.60 substantial strength of agreement between tests.

AUC: area under curve from ROC curve analyses; PPV: positive predictive value; NPV: negative predictive value.

**Table 3:** Concordance (kappa values) between independence assessment tools at different cut-off values.

	Established cut-offs		Optimal cut-offs		
	HY > 3.0 *N* = 14	SE < 80 *N* = 40	HY > 2.0 *N* = 67	SE < 90 *N* = 80	BI < 20 *N* = 61
HY stage > 3.0	—	0.45	0.23	0.17	0.27
SE score < 80	0.45	—	0.52	0.50	0.56
HY stage > 2.0	0.23	0.52	—	0.48	0.40
SE score < 90	0.17	0.50	0.48	—	0.46
BI score < 20	0.27	0.56	0.40	0.46	—

Kappa values exceeding 0.00 represent slight, 0.20 fair, 0.40 moderate, and 0.60 substantial strength of agreement between tests.

HY: Hoehn and Yahr stage; SE: Schwab and England ADL scale score; BI: Barthel ADL index.
